# Therapeutic potential of NR4A1 in cancer: Focus on metabolism

**DOI:** 10.3389/fonc.2022.972984

**Published:** 2022-08-16

**Authors:** Shan Deng, Bo Chen, Jiege Huo, Xin Liu

**Affiliations:** ^1^ Third School of Clinical Medicine, Nanjing University of Chinese Medicine, Nanjing, China; ^2^ Materials Science and Devices Institute, Suzhou University of Science and Technology, Suzhou, China; ^3^ Department of Orthopedics, Nanjing Lishui Hospital of Traditional Chinese Medicine, Nanjing, China

**Keywords:** metabolic reprogramming, NR4A1, cancers, signaling pathways, tumor cells

## Abstract

Metabolic reprogramming is a vital hallmark of cancer, and it provides the necessary energy and biological materials to support the continuous proliferation and survival of tumor cells. NR4A1 is belonging to nuclear subfamily 4 (NR4A) receptors. NR4A1 plays diverse roles in many tumors, including melanoma, colorectal cancer, breast cancer, and hepatocellular cancer, to regulate cell growth, apoptosis, metastasis. Recent reports shown that NR4A1 exhibits unique metabolic regulating effects in cancers. This receptor was first found to mediate glycolysis *via* key enzymes glucose transporters (GLUTs), hexokinase 2 (HK2), fructose phosphate kinase (PFK), and pyruvate kinase (PK). Then its functions extended to fatty acid synthesis by modulating CD36, fatty acid-binding proteins (FABPs), sterol regulatory element-binding protein 1 (SREBP1), glutamine by Myc, mammalian target of rapamycin (mTOR), and hypoxia-inducible factors alpha (HIF-1α), respectively. In addition, NR4A1 is involving in amino acid metabolism and tumor immunity by metabolic processes. More and more NR4A1 ligands are found to participate in tumor metabolic reprogramming, suggesting that regulating NR4A1 by novel ligands is a promising approach to alter metabolism signaling pathways in cancer therapy. Basic on this, this review highlighted the diverse metabolic roles of NR4A1 in cancers, which provides vital references for the clinical application.

## Introduction

Metabolic reprogramming is an important characteristic of tumor cells, which can provide energy and multiple substrates for biosynthesis to support cancer cells’ rapid proliferation and survival (1). Furthermore, the malignant transformation, invasion, and metastasis of tumor cells also need metabolic reprogramming. The metabolic pathways include glycolysis, glutamine metabolism, fatty acid metabolism, nucleic acid, and amino acid metabolism (2). In the 1920s, Otto Warburg first reported the Warburg effect, which was once considered the main metabolic pathway in cancer cells. Nowadays, this effect is defined as aerobic glycolysis, which can metabolize glucose into lactate and provides Adenosine triphosphate (ATP) for cancer cell survival. By downregulating aerobic glycolysis via the c-Myc signaling pathway, the proliferation of hepatocellular carcinoma (HCC) was inhibited, and their apoptosis was induced (3). Besides, glutamine has been reported to contribute to tricarboxylic acid (TCA) cycle metabolites. The “glutamine addiction” is a vital metabolic feature to support the rapid proliferation of cancer cells. In breast cancer, some polyphenols, such as catechin, delphinidin, and kaempferol, exhibit an anti-proliferative effect by inhibiting alanine, serine, cysteine transporter 2 (ASCT2) and decreasing total and Na+-dependent 3H-glutamine uptake (4). The fatty acid is the key competitor of the cell membrane and stores energy and acts as the secondary messengers. Thus, fatty acid synthesis (FSAN) is vital for transporting intracellular signal transduction and tumor cell proliferation, differentiation, migration, survival, and apoptosis (5). Fan et al. (6) reported that α-linolenic acid could inhibit osteosarcoma cell proliferation and metastasis by suppressing FASN expression. By blocking fatty acid enzymes hexokinase 2 (HK2) or acyl-CoA synthetase long-chain family member 4 (ACSL4), acetyl-CoA accumulation decreased, leading to a suppressed fatty acid β-oxidation activity. These results effectively inhibit liver cancer growth (7). In order to meet the infinite proliferation in tumor cells, transcription and replication activities are more frequent, so the nucleotides and amino acids are enhanced. Buel et al. (8) reported the crosstalk between amino acid and mTORC1, which can regulate tumor cell fate through the Rag-GTPase pathway. As mentioned above, cancer metabolism is controlled by many factors, including genes, enzymes, and signaling pathways; therefore, exploring novel targets of metabolic reprogramming provides enormous opportunities to regulate tumor cells fate.

**Figure 1:**
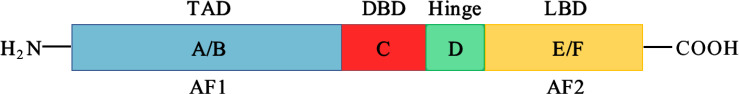
The structure of NR4A receptors. NR4A structure has a N-terminal domain containing AF-1, and C-terminal domain with AF-2, they flank a DNA-binding domain (DBD) and a hinge region.

In recent years, metabolism has been widely observed during cancer development; NR subfamily 4 (NR4A) receptors are considered the mediators in controlling this metabolic hallmark of tumors. NR4A family receptors are one of 48 human nuclear receptors that act as transcription factors to regulate many cell processes. NR4A nuclear receptors include NR4A1 (NUR77), NR4A2 (NURR1), and NR4A3 (NOR-1), showing similar structures which consist of a DNA-binding domain (DBD), a C-terminal ligand-binding domain (LBD), and an N-terminal transactivation domain (TAD). The TAD contains a ligand-independent activation function 1 (AF-1), responsible for interacting and regulating the activity of transcription factors. The DBD in the middle can specifically interact with DNA sequences known as NGFI-B response element (NBRE) and Nur-responsive element (NurRE); there is over 90% sequence homology in DBD of NR4A receptors (9). The last part, LBD, contains a ligand-dependent activation function 2 (AF-2), which can recognize corresponding ligands to ensure the transcriptional activity (10–12) (**Figure 1**). Although these receptors share a typical structure, about 60% of the sequences in the LBD region are conserved, while the sequence of the activation domain changes greatly. Because the large hydrophobic residues occupy the binding pocket space, there has no progress in identifying endogenous ligands so far. Recent reports suggest that the NR4A family receptors may bind to unsaturated fatty acids in the LBD to exhibit regulation of metabolism (13). For example, in breast cancer, NR4A1-NR4A3 regulates glycolysis to participate in cell progression (14). The NR4A receptors are also associated with the activation of T cell, which involving in cancer immunotherapy (15). From the literature, increasing evidence proves that among these NR4A receptors, NR4A1 shows more metabolic functions in cancers, such as regulating glycolysis and exhibiting activities in fatty acid synthesis, glutamine, and amino acid metabolism. This paper summarizes the metabolism roles of NR4A1 in the tumor.

## Identification and regulation of NR4A1

**Figure 2:**
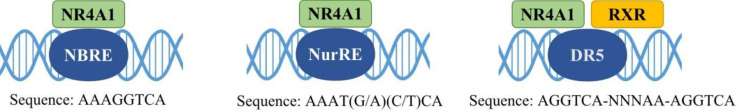
Interactions of NR4A1 with different elements. NR4A1 activates target gene expression through binding with NBRE, NurRE, and a DR5 motif (with RXR), respectively.

**Figure 3:**
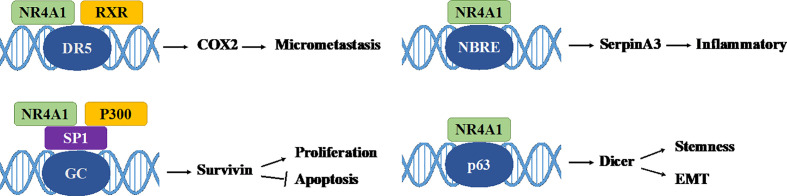
Some genomic effects of NR4A1. NR4A1 can bind to different genes promoter to involve in cancer progression. GC: GC-rich promoter regions of Survivin.

Nuclear receptor 4A1 (NR4A1, also called Nur77, NGFIB, TR3) is one of the NR4A subfamily transcription factors, which was firstly identified in mouse fibroblasts in 1988 (16). Next year, Chang (17) isolated NR4A1 from a human prostate lambda gt11 cDNA library. Then it is found in various tissues and cells, including cancer cells. NR4A1 is an immediate gene induced by stress, cytokines, growth factor, glucose, fatty acids, or other stimuli (18–21). NR4A1 plays diverse roles in many physiological and pathological processes, for example cell survival, apoptosis, differentiation, cell cycle, inflammation, immunity, and metabolism (22–26). NR4A1 can bind to DNA in three ways to regulate the expression of target genes: (1) it can form the response element NBRE (sequence: AAAGGTCA); (2) it binds to the NurRE element (AAAT(G/A)(C/T)CA, which are related to the NBRE) in the form of homodimer or heterodimer formed with other members of the family; (3) NR4A1 and retinoid X receptors (RXRs) form heterodimers and then binds to the DR5 response element to produce transcriptional activation (sequence: AGGTCA-NNNAA-AGGTCA) (27) (**Figure 2**). Because of the specific structure, NR4A1 can directly affect the target genes promoter to exhibit transcriptional activity. For example, in prostate cancer, prostaglandin E2 (PGE2) activates NR4A-RXR heterodimer to enhance micrometastasis; this effect can be reversed by cyclooxygenase 2 (COX2) inhibitor in cancer suppression (28). NR4A1 also complexes with Sp1 and p300 on the region of survivin promoter to increase pancreatic cancer cell proliferation and decrease apoptosis (29). In inflammatory diseases, NR4A1 could regulate SerpinA3 through the NBRE in its promoter region (30). NR4A1 transcriptionally inhibits the expression of Dicer to activate downstream Akt/mTORC1 signaling, thereby inducing colon cancer epithelial-to-mesenchymal transition (EMT) (31) (**Figure 3**).

**Figure 4:**
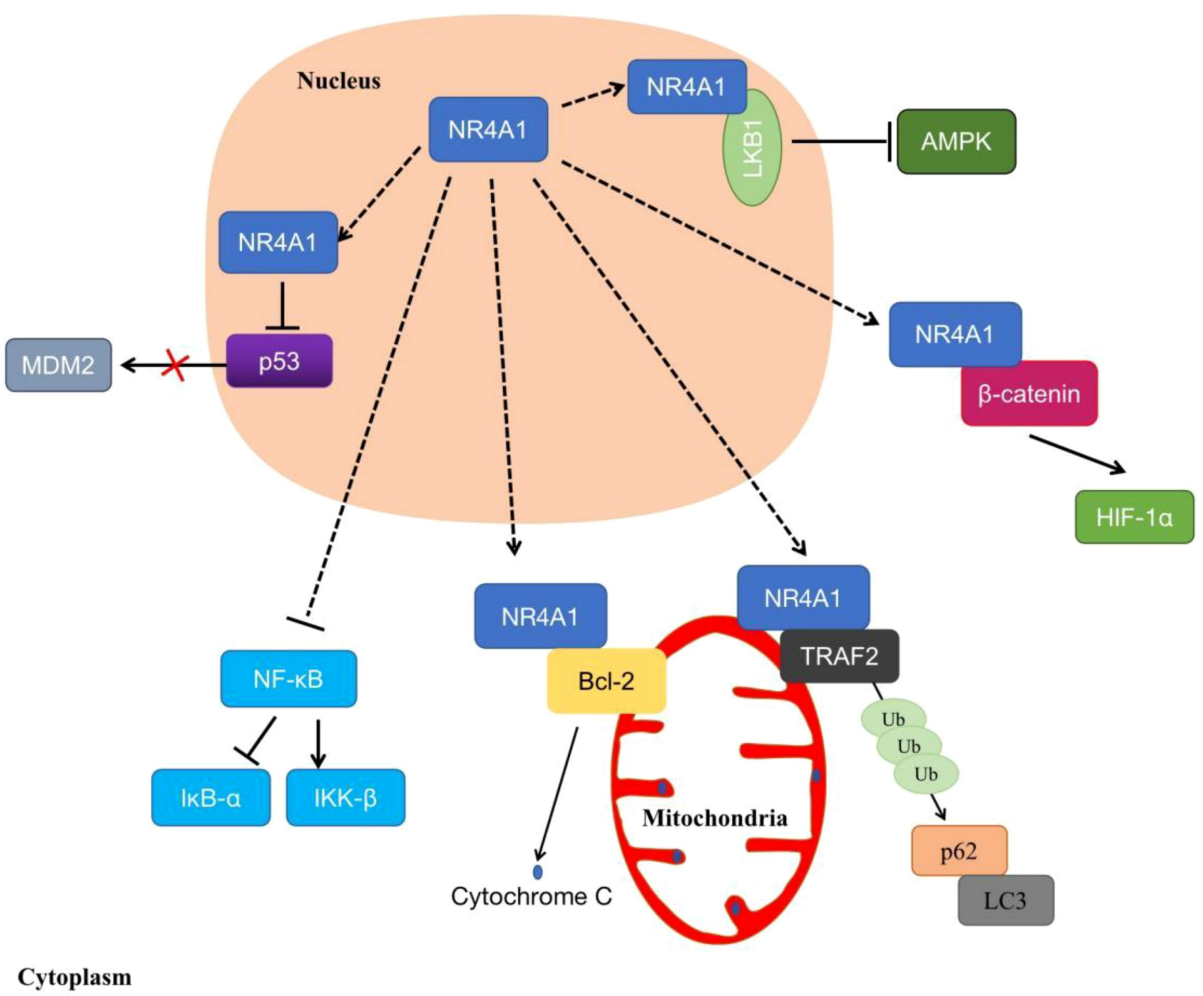
The non-genomic regulations of NR4A1. NR4A1 can affect kinds of biological processes through binding to LKB1, β-catenin, Bcl-2, MDM2, TRAF2, or blocking NF-κB.

In addition, increasing evidence indicates that NR4A1 displays non-genomic functions to affect cell biological processes. NR4A1 exerts non-genomic activities by translocating from the nucleus to mitochondria, or endoplasmic reticulum (ER), which triggers apoptosis or autophagy (32). Studies have shown that the overexpressed NR4A1 can activate the Wnt/β-catenin signaling pathway to enhance colon tumor growth, colony formation, and migration (33). It also moves to the cytoplasm to stimulate the dysregulation of β-catenin and the stabilization of HIF-1α under normoxia (34). In mitochondrial, NR4A1 translocated from the nucleus and bound to Bcl-2, converts Bcl-2 to a pro-apoptotic protein, then induces cytochrome C release and apoptosis (35). Another report showed that NR4A1 could induce MDM2 ubiquitination and degradation by blocking p53 acetylation, this effect can enhance p53-depended apoptosis (36). Furthermore, NR4A1 interacts, and blocks binds and sequesters Liver kinase B1 (LKB1) in the nucleus, then releases and shuttles LKB1 to the cytoplasm, thereby attenuating AMP-activated protein kinase (AMPK) activation to treat metabolic diseases such as streptozotocin-induced diabetes (37). In osteoclast, knockout of NR4A1 can promote the differentiation of RAW264.7 by activating the NF-κB signaling pathway, in order to decrease the expression of IκB-α and induce IKK-β (23). Additionally, NR4A1 translocated from nucleus to mitochondria, then interacted with tumor necrosis factor receptor-associated factor 2 (TRAF2), leading to TRAF2 ubiquitination. NR4A1 also interacted with p62/SQSTM1 to sensitize cells to autophagy (38) (**Figure 4**).

## Metabolism roles of NR4A1 in tumor

NR4A1 is widely involved in the metabolism of tumors, including glucose metabolism, glutamine metabolism, fatty acid metabolism, and amino acid metabolism (39–42).

### NR4A1 and glucose metabolism

Glucose metabolism is the key source to provide metabolic carbon in cells. There are three main ways of glucose metabolism: aerobic oxidation, glycolysis and pentose phosphate pathways. Normally, cells uptake glucose by glucose transporters (GLUTs), then it enters the glycolysis process with the action of hexokinase (HK), fructose phosphate kinase (PFK), and pyruvate kinase (PK) under normal oxygen conditions to produce pyruvate. However, uncontrolled proliferation is a crucial characteristic of cancer. Tumor cells alter their glucose metabolism patter to an efficient aerobic glycolysis rate to sustain vigorous proliferation and other biological activities. In this process, the activities and expression levels of GLUT and glycolytic rate-limiting enzymes such as HK, PFK, and PK were significantly up-regulated to improve glucose uptake, which is called the “Warburg effect” (43). This effect not only provides the energy for tumor cell survival, but also provides biosynthetic raw materials for other metabolic pathways, including the tricarboxylic acid (TCA) cycle, hexosamine pathway, pentose phosphate pathway, glycogen synthesis, and serine biosynthesis pathway (44).

Recently, it has been progressively realized that NR4A1 plays diverse roles in glucose metabolic regulation. GLUTs facilitate the transport of glucose from extracellular to the cellar membrane. Overexpressed NR4A1 has been reported to upregulate GLUT4 production to increase glucose oxidation and glycogen synthesis in muscle L6 cells. NR4A1 also changes the activity of several key glycolytic enzymes; for example, NR4A1 upregulates the expression of HK and PFK in rat muscle cells (45). In HFD-induces obese mice, inhibition of NR4A1 by siRNA could modulate the key rate-limiting enzyme HK2, leading to the disturbed glucose metabolism homeostasis in mice cardiac (46). Furthermore, bis-indole-derived NR4A1 ligands enhanced the accumulation of GLUT4 in the cell membrane and the overall glucose uptake in muscle cells in diabetes (47). NR4A1 is considered a promising therapeutic target for metabolic syndromes.

In addition, the paradoxical roles of NR4A1 in regulating glucose metabolism in cancer were investigated. In hepatocellular carcinoma, low expression of NR4A1 was observed, promoting HCC development. NR4A1 can inhibit glycolysis and elevate gluconeogenesis by interacting with and suppressing the rate-limiting enzyme phosphoenolpyruvate carboxykinase (PEPCK1), leading to ATP depletion and an arrest of cell growth (48). Another research reported that by binding to the promoter of WAP four-disulfide core domain 21 pseudogene (WFDC21P), NR4A1 also inhibited two key glycolysis enzymes, the platelet-type PFK (PFKP) and the M2 isoform of pyruvate kinase (PKM2), to suppress the HCC cell proliferation and tumor metastasis (49). Furthermore, in acute promyelocytic leukemia (APL) cells, silencing NR4A1 can activate glycolytic transporter GLUT1 and decrease the expression of TIGAR (TP53-induced glycolysis and apoptosis regulator) to induce APL development (50). Cytosporone B (CsnB) is an NR4A1 agonist; it induced tumor cell apoptosis and inhibited tumor growth in C57 mice via translocating NR4A1 to mitochondrial to cause cytochrome C release. CsnB also induced gluconeogenesis-related genes, resulting in elevated of blood glucose levels in tumors (51).

In contrast, NR4A1 is overexpressed in many other human malignant tumors, for example, pancreatic cancer, colorectal cancer, and breast cancer. Several studies have revealed that hypoxic exposure results in increased HIF-1α protein stabilization, which has been implicated in promoting the glycolysis of tumor cells. This response can be regulated by NR4A1 through repressing MDM2 expression, suggesting the enhancement of glycolysis induced by HIF-1α was partially attributed to NR4A1 upregulation (52, 53). In colorectal cancer cells, Dong et al. (54, 55) reported the relationship between enhanced glycolysis and the aberrant activation of β-catenin, while our previous study confirmed that β-catenin and NR4A1 could form a mutually feedback control circuit to promote CRC invasion, demonstrating that NR4A1 may be involved in the glycolysis in colorectal cancer. These findings underscore the regulation of NR4A1 on glucose oxidation and glycogen synthesis, indicating that the impact of NR4A1 on glucose metabolism is complex and cell-dependent.

### NR4A1 and glutamine metabolism

Glutamine metabolism is dysregulated in a variety of solid tumor cells, and it is indispensable for cancer cell proliferation. Depletion of glutamine can promote EMT and metastasis, overcome tumor immune evasion (56–58). Therefore, glutamine has become a very attractive target for tumor anti-metabolic therapy. To be better utilized by cells, glutamine is transported into cells through specific transporters and converted into glutamate under the action of glutaminase to enter the TCA cycle and provide energy for the growth and development of tumor cells. Glutamine enters cells via the solute carrier family (SLC) transporters, including SLC1A5 and SLC7A8, which are overexpressed in many cancers. These Na+-independent neutral amino acid transporters can activate mTOR signaling and are controlled by Myc (59). In 2009, Gao et al. (60) reported that Myc inhibits glutamine metabolism by suppressing miR-23a/b expression to generate energy for proliferating cancer cells. In ovarian cancer, miR-145 decreased glutamine metabolism through targeting c-Myc via activating glutaminase 1 (GLS1) transcription expression (61). In pancreatic cancer, the nuclear translocation of β-catenin can increase c-Myc expression, resulting in a rise in glutamine uptake and glutamate release (62). On the other hand, a study indicated that NR4A1 acts as a β-catenin mediator to allow β-catenin to escape degradation in HCC (63). Meanwhile, our previous study reported a positive NR4A1-β-catenin feed-forward loop in cancers (31, 55). Another study reported that NR4A1 inhibition decreased the levels of β-catenin and c-Myc (64); thus, it is reasonable to speculate that NR4A1 may participate in glutamine metabolism through β-catenin/Myc signaling pathway. In terms of mTOR, glutamine upregulated the activity of glutaminase (GLS) and glutamate dehydrogenase (GDH) by inducing mTOR upregulation. This effect can be reversed by mTOR inhibitor rapamycin, leading to a decrease of glutamine-induced cell proliferation in ovarian cancer (65). NR4A1 can regulate mTOR signaling, and knockdown of NR4A1 inhibits mTOR through reactive oxygen species-dependent activation of AMPK (66, 67), so NR4A1 may be involved in glutamine metabolism via mTOR regulation.

Furthermore, reduced oxygen supply increases GLS1 mRNA and protein expression due to transcriptional activation of HIF-1, accelerates glutamine metabolism, and is conducive to the growth, invasion, migration, as well as metastasis in colorectal cancer (68). NR4A1 is an important regulator of HIF-1. The relationship between NR4A1 and HIF-1 has been shown in many tumors (53, 69). In renal cell carcinoma, NR4A1 stabilized and transactivated HIF-1α. Moreover, NR4A1 is highly expressed in acute myeloid leukemia; when truncated protein-encoding for part of the N-terminal domain of NR4A1, the NR4A1 transcript variant still maintains the stability and activity of HIF-1α (70). On the other hand, HIF-1α activated NR4A1 by binding to the putative HIF responsive element in the NR4A1 promoter, then upregulating the expression of NR4A1 (71). Under chronic hypoxia conditions, NR4A1 has low expression in non-small cell lung cancer (NSCLC) cells by the mediation of HIF-1α, involved in hypoxia-induced apoptosis resistance (72). Therefore, NR4A1 and HIF-1α can form an interaction circulus, influencing each other. Since HIF-1 is a vital regulator in glutamine metabolism, NR4A1 is likely to become a potential target of tumor glutamine metabolism.

### NR4A1 and fatty acid metabolism

Lipids are classified as fatty acids, cholesterol, phospholipids, or triacylglycerides, major components of cell membranes. Lipids are widely contributed to energy sources, signaling molecules, and second messengers. As an important component of various lipids, fatty acid synthase (FASN) plays an irreplaceable role in cell proliferation and survival. Various raw materials for fatty acid synthesis synthesize fatty acids from scratch under the catalysis of enzymes such as ATP citrate lyase (ACLY), acetyl CoA carboxylase (ACC), and fatty acid synthase (FAS). Accumulating evidence has shown that dysregulation in lipid metabolism is one of the most abnormal metabolic changes in tumor cells, while the enhancement of de novo fatty acid synthesis is the main manifestation of lipometabolic reprogramming in tumor cells. The FASN process leads to the increased expression of a variety of key enzymes, mainly ACLY, ACC, and FAS (73), which affect multiple aspects of carcinogenesis, such as cell proliferation, differentiation and cell cycle (74).

Cancers drive fatty acid mainly from exogenously microenvironment or endogenously through de novo synthesis by FASN. The most well-characterized transporters include CD36, solute carrier protein family 27 (SCL27), and fatty acid-binding proteins (FABPs). Among them, CD36 is reported to be highly expressed in various malignancies, including breast cancer, ovarian cancer, and gastric cancer (75–77). SCL27 family has six members (SCL27 A1-A6) associated with tumor fatty acid uptake (78, 79). FABPs consists of 12 family members, and they can transport lipid to cellular mitochondria, nuclei, and so on (80). And they are frequently found to be highly expressed in bladder cancer, prostate cancer, and renal cell carcinoma (81, 82).

In the early decade, numerous studies explored the complex roles of NR4A1 in regulating fatty acid metabolism in normal tissues, including liver, skeletal muscle, and adipose. For example, Wang’s team reported (83) that NR4A1 could specifically bind to LKB1 in the nucleus and prevent the translocation of LKB1 to the cytosol. This interaction between NR4A1 and LKB1 can be broken by antagonist TPMA, promoting of AMPKα phosphorylation and activating downstream fatty acid enzymes like ACC and CPT1A to inhibit fatty acids synthesis in primary hepatocytes. Sterol regulatory element-binding protein 1 c (SREBP1c) is a well-established transcription factor to regulate FASN (84), to regulate hepatic lipid metabolism, NR4A1 decreased SREBP1c expression by reducing its target genes stearoyl-coA desaturase-1(SCD1), mitochondrial glycerol-3-phosphate acyltransferase (GPAT), and FASN (85). In skeletal muscle cells, attenuation of NR4A1 expression decreased lipolysis by inhibiting beta-AR and its downstream CD36, adiponectin receptor 2, and caveolin-3 expressions (86). Like in liver cells, Jung’s team reported (86, 87) that the interaction of NR4A1 and AMPKα in inhibiting adipogenesis *in vitro* and *in vivo*.

**Figure 5:**
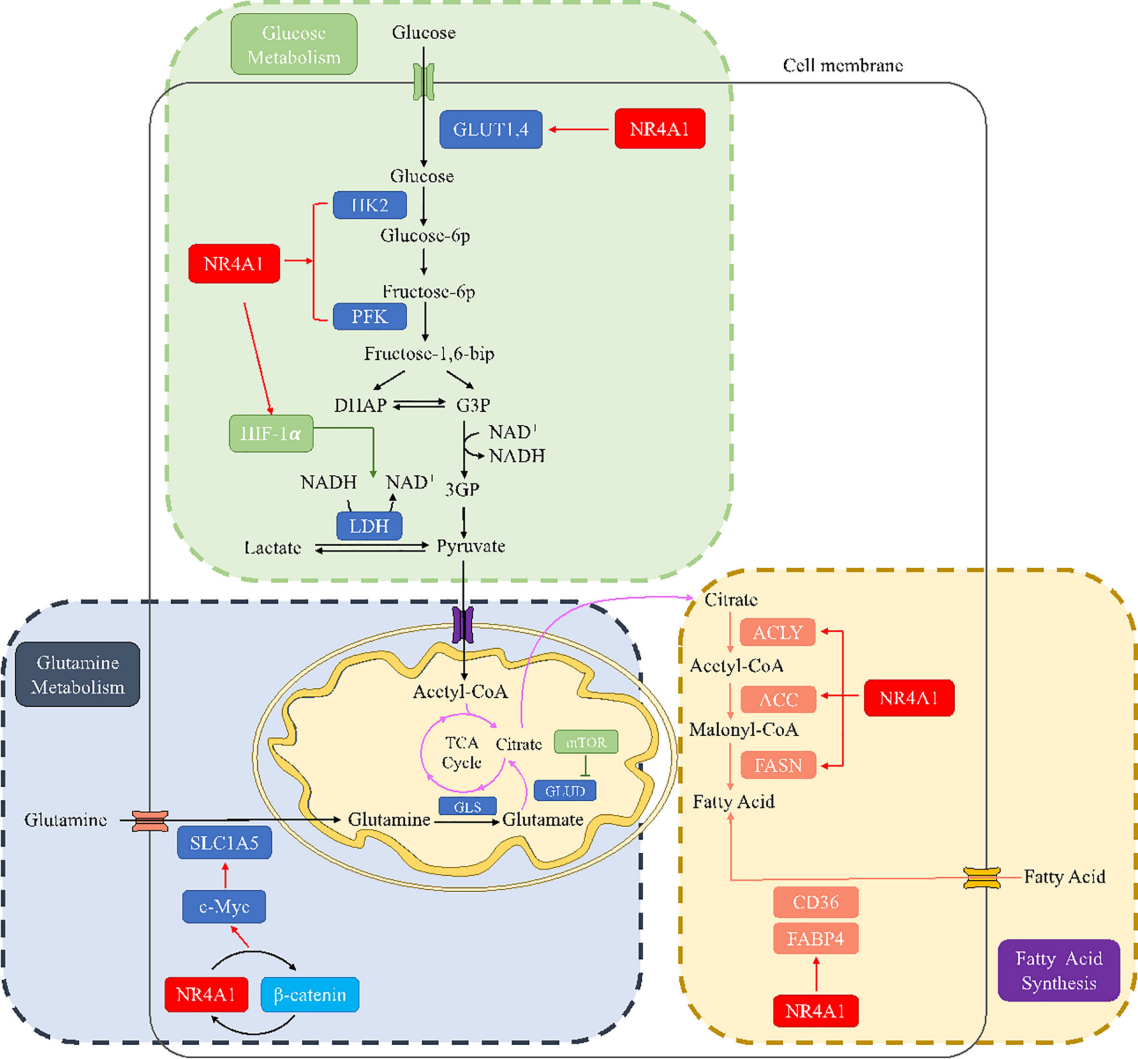
The metabolic regulation of NR4A1 in glucose, glutamine, and fatty acid metabolism. In glucose metabolism, NR4A1 can regulate glucose transporter GLUT1 and 4, key enzymes HK and PFK, as well as HIF-1α. NR4A1 mediates glutamine metabolism by regulating SLC1A5 *via* c-Myc. NR4A1 can regulate the fatty acid synthesis of major enzymes ACLY, ACC, and FASN, and it also participates in fatty acid uptake through CD36 and FABP4.

Recently, the lipid metabolic roles of NR4A1 attracted more and more attention in tumor progression, especially fatty acid metabolism. Fatty acid metabolism includes fatty acid synthesis and fatty acid oxidation (FAO). NR4A1 is thought to participate in fatty acid uptake and oxidation to affect cancer cell fate. Fatty acid oxidation provides the ATP and NADPH to overcome metabolic stress. To assess the role of NR4A1 in cancers, a recent study reported that NR4A1 is required in melanoma cells to protecting FAO. The overexpressed NR4A1 can bind to and activate the rate-limiting enzyme trifunctional protein β (TPβ) to maintain ATP and NADPH levels and prevent ROS increase and melanoma cell death. NR4A1 regulated the linkage FAO-NADPH-ROS during metabolic stress to target melanoma (88). Holla et al. (89) reported the pro-oncogenic effect of NR4A1 in regulating the fatty acid oxidation pathway in colon cancer. A high level of PGE2 induced-NR4A2 was reported to bind to NR4A1-binding response elements (NBRE), which can recruit and induce the expression of four genes related to fatty acid metabolism: acyl-CoA oxidase (ACOX), carnitine palmitoyltransferase 1B (CPT1M), fatty acid-binding protein-2 (FABP2) and FABP4. A novel study reported that peroxisome proliferator-activated receptor-γ (PPARγ) acts as an antagonist of NR4A1 and can ubiquitination and degradation of NR4A1 through ubiquitin enzyme tripartite motif 13 (TRIM13); this process interferes with the interaction of NR4A1 and SWI/SNF complex, and recruit to the promoter of fatty acid transporters CD36 and FABP4 to inhibit their transcription, which blocked fatty acid uptake to suppress cancer cell proliferation (41) (**Figure 5**).

Apart from fatty acid synthesis, NR4A1 also participates in cholesterol metabolite, Dendrogenin A (DDA) is identified as a cholesterol metabolite in mammal cells. There has a complemental effect between cancer cells and DDA; by binding to the liver X receptor (LXR), DDA can activate NR4A1 expression to exhibit an anti-tumor effect on breast cancer and melanoma (90). In acute myeloid leukemia, DDA also partly activates LXR to increase NR4A1, further inhibiting the expression level of cholesterol biosynthesizing enzyme 3β-hydroxysterol-Δ8,7-isomerase (D8D7I), leading to cancer autophagy induction (91). In HepG2 cells, downregulation of NR4A1 induced an increase in total cholesterol (TCHO) levels, low-density lipoprotein receptor (LDLR), and HMGCoA reductase (HMGCR) levels are also increased following the inhibition of NR4A1, suggesting NR4A1 is capable of reducing hepatic cholesterol based on lipid overloading. This evidence is proved that the effect of NR4A1 in regulating lipid metabolism in cancer growth and proliferation.

### NR4A1 and amino acid metabolism

**Figure 6:**
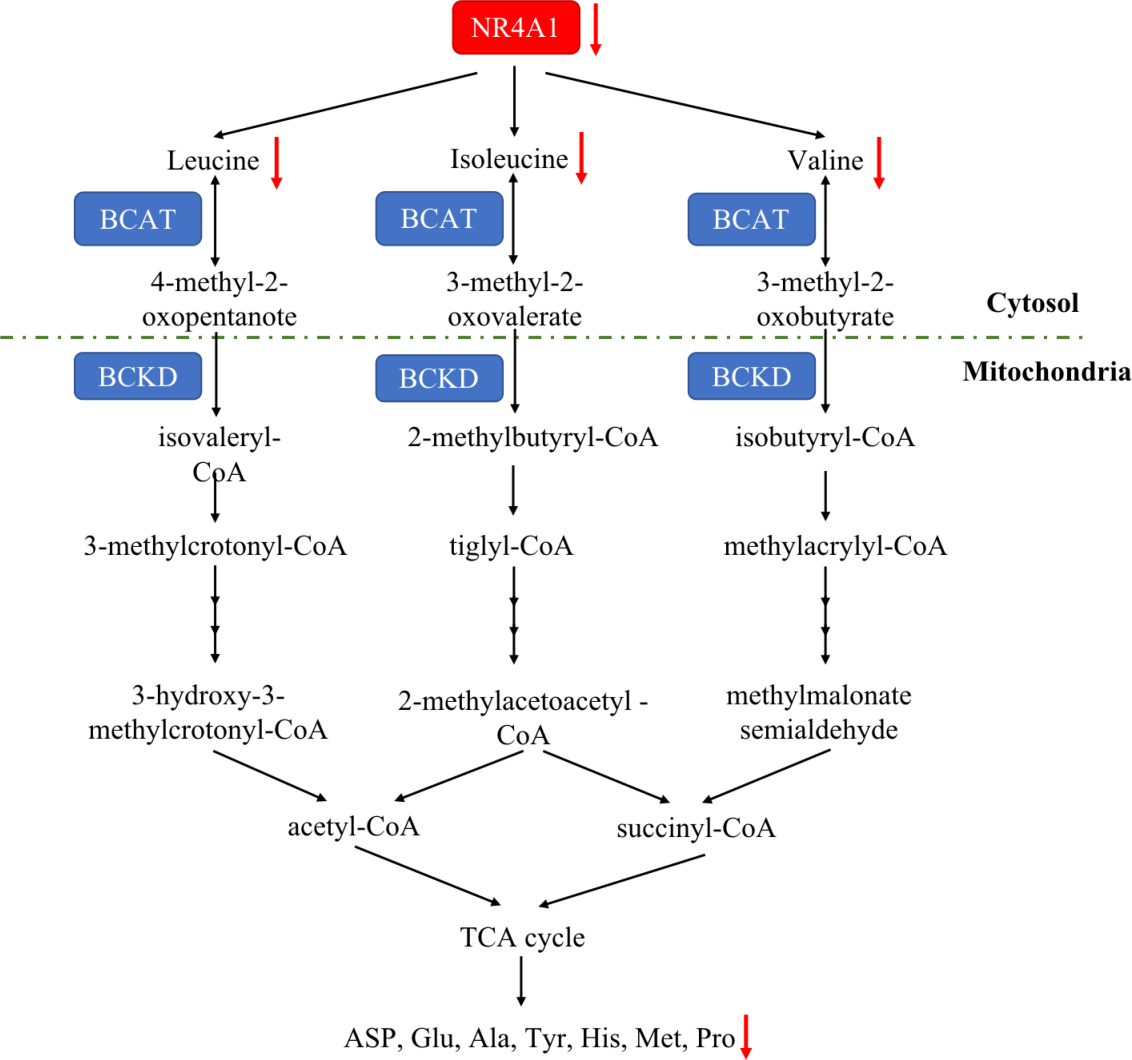
The amino acid metabolic pathway of NR4A1. In zebrafish larva, NR4A1 deficiency can decrease key branched-chain amino acids Val, Leu, and Ile, leading to the inhibition of ASP, Glu, Ala, Tyr, His, Met and Pro.

Amino acid is essential for mammalian cells as the substrate for new protein synthesis. However, to drive the continuous proliferation of cancer cells, an abundant supply of amino acids is observed (92). A novel reports demonstrated that amino acid deficiency (AAD) could activate myocyte enhancer factor 2D (MEF2D) and induce the expression of NR4A1, which mediated reticulophagy to maintain intracellular amino acid levels (93). Although amino acid deficiency induces NR4A1, there are rare study focusing on the connection between NR4A1 and amino acid metabolism. Almost 20 years ago, Li and colleagues found (94) that changing the DNA-binding site of NR4A1 at Ser354 with negatively charged amino acids, such as Asp or Glu, can significantly decrease the NR4A1 transactivation activities. A most recent study from Xu et al. (21) first indicated the role of NR4A1 in regulating amino acids. They observed total amino acids compositions and found valine (Val), leucine (Leu), and isoleucine (Ile) were all decreased as well as many other amino acids, including aspartic acid (ASP), glutamic acid (Glu), alanine (Ala), tyrosine (Tyr), histidine (His), methionine (Met), proline (Pro), and so on in NR4A1−/− zebrafish larva (**Figure 6**).

## NR4A1 and tumor microenvironment

**Figure 7:**
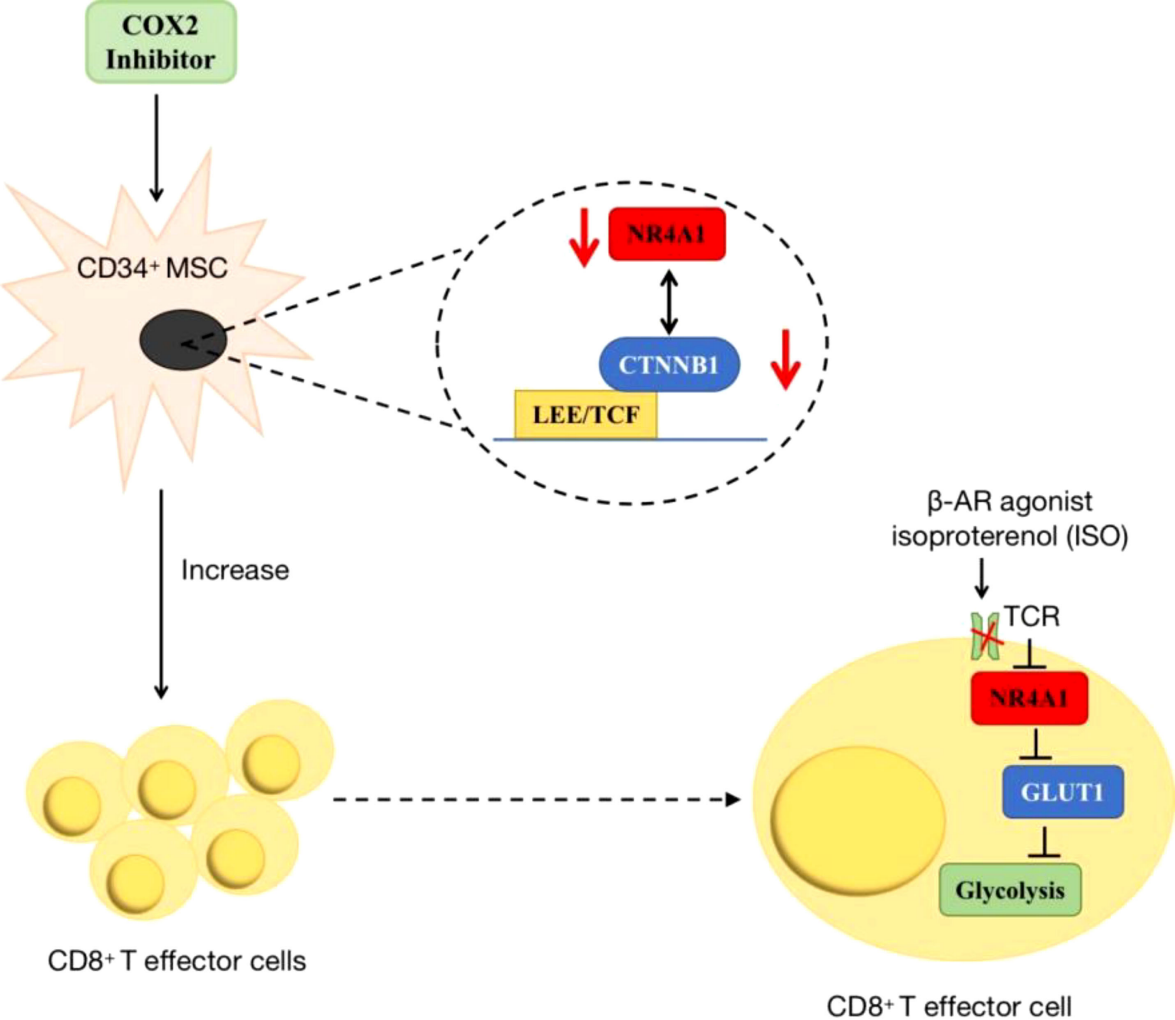
The roles of NR4A1 in regulating T cell. In AML, the inhibition of COX2/NR4A1/CTNNB1 signaling pathway can increase the produce of CD8+ T cell to rescue anti-cancer immunity. In melanoma, β-AR agonist inhibits NR4A1 and its downstream glycolysis to mediate CD8+T cell activity.

Additionally, NR4A1 also participated in cancer immunity by regulating metabolic pathways. In acute myeloid leukaemia (AML), researchers found COX2 inhibition dramatically decreased NR4A1 transcription and the WNT signaling pathway. In AML-mesenchymal stromal cells (MSCs)-CD34+ cells co-cultured system, this a novel COX2/NR4A1/CTNNB1 axis increased leukaemia-reactive T-effector cells and rescued cellular metabolism and anti-leukaemia immunity (95). In the melanoma tumor microenvironment, T-cell receptor (TCR) signaling can trigger its downstream NR4A1 expression, so using NR4A1-GFP indicated that blocking β-AR signaling increased metabolic reprogramming of CD8+ T-cell activation via TCR signaling. This impairment of β-AR on TCR signaling occurs through GLUT-1 downregulation and subsequent increase of glycolysis (96) (**Figure 7**).

## Potential for targeting NR4A1

**Figure 8:**
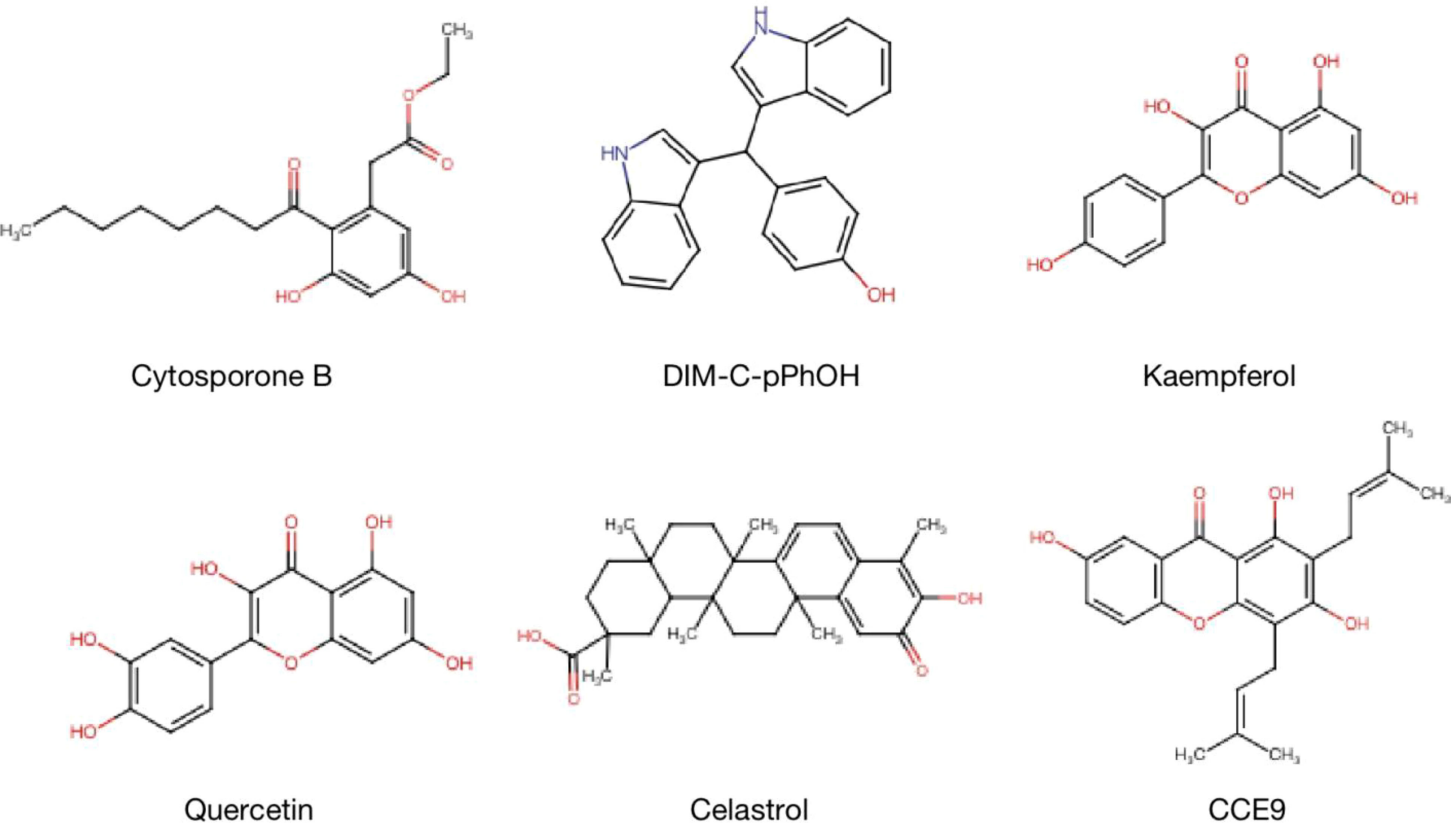
Structure of NR4A1 agonists or antagonists. Structure of NR4A1 inducer Cytosporone B, and inhibitors DIM-C-pPhOH, Kaempferol, Quercetin, Celastrol, CCE9.

The expression and functions of NR4A1 in cancer metabolism are emerging as a promising area in treating and preventing human cancer malignant evolvement. Developments on mechanisms of NR4A1 silencing or strategies for its activity are leading to the explore of novel therapeutic agents. For example, CsnB is the first described NR4A1 agonist; multiple studies have indicated the CsnB can bind to LBD of NR4A1 and modulate NR4A1 nuclear export to mitochondria, causing Cyto-C release and apoptosis (51). Additionally, CsnB also acts as a candidate to downregulate CD36/FABP4 expression, leading to the inhibition of fatty acid uptake and consequent breast cancer cell proliferation in NR4A1-dependent manner (41). A class of Bisindole-derived (CDIMs) NR4A1 antagonists, such as 1,1-bis(3’-indolyl)-1-(p-hydroxyphenyl) methane (DIM-C-pPhOH), can decrease the expression of NR4A1 in breast, lung, and liver cancer cells to inhibit tumor growth, EMT and stemness (97, 98). Additionally, some natural compounds also act as NR4A1 ligands to exhibit an anti-tumor effect. Kaempferol and Quercetin are flavonoid compounds; they bind to NR4A1 and inhibit NR4A1-dependent transactivation by decreasing PAX3-FOXO1-G9a and mTOR signaling to suppress RMS cell growth (64). 1,3,7-trihydroxy-2,4-diprenylxanthone (CCE9) is a xanthone compound that induces the expression of NR4A1 and the interaction of NR4A1 and Bcl-2, leading to increased apoptosis through p38α/MAPK signaling pathway (99). Celastrol has a potent anti-inflammation effect by binding to NR4A1 and inducing NR4A1 to transport to mitochondria, resulting in sensitivity to autophagy (38). (**Table 1** and **Figure 8**).

**Table 1:** NR4A1 ligands.

Type	Name	Target	Applications	Ref
Inducer	Cytosporone B	Cyto-C	Breast cancer Colon cancer Lung cancer Bladder cancer	(41, 72, 100, 101)
Inhibitor	DIM-C-pPhOH		Breast cancer Rhabdomyosarcoma Pancreatic cancer	(102–104)
	Kaempferol	mTOR	Rhabdomyosarcoma	(64)
	Quercetin	mTOR	Rhabdomyosarcoma	(64)
	CCE9	p38α MAPK Bcl-2	Cervical cancer Liver cancer	(99)
	Celastrol	p62	Cervical cancer	(38)

## Discussion

Aberrant metabolism reprogramming is a core feature of the tumor; increased cancer metabolism, such as fatty acid synthesis, glycolysis, plays vital roles in tumor proliferation, metastasis, and multidrug resistance. Hence, developing novel therapeutic methods and drug targets are required. Accumulating evidence proves that NR4A1 implicated metabolic processes in regulating various diseases, including obesity, atherosclerosis, liver disorders, and diabetes.

NR4A1 is an orphan nuclear receptor that exhibits pro-oncogene or anti-cancer effects in different cancers. For example, in colorectal cancer, overexpressed NR4A1 promoted cancer cell growth, epithelial-mesenchymal transition (EMT), and cancer stem-like cells (CSCs) properties. However, in HCC, NR4A1 is low expressed, upregulating NR4A1 by CsnB or other compounds, such as 4-(quinoline-4-amino), can inhibit tumor cell growth *in vitro* and *in vivo* (105). In terms of metabolism, it has been found that targeting NR4A1 can regulate glycolytic key enzymes GLUT4, HK2, and PFK in the liver and muscle cells to target metabolic syndromes. Although the metabolic roles of NR4A1 have been reported, the diverse effects in cancer metabolic reprogramming have not been delineated. This review links NR4A1 to metabolic processes in cancers. By altering glucose metabolism, NR4A1 depleted ATP and induced cell cycle arrest in HCC. NR4A1 also inhibited glycolysis enzymes PFKP and PKM2 to block HCC metastasis. The NR4A1 mediator CsnB induced tumor cell apoptosis; this suppressive function of CsnB is associated with the translocation of NR4A1 from the nucleus to mitochondria to release the cytochrome C-depended Bcl-2 apoptotic pathway. Paradoxically, in colorectal, pancreatic, and breast cancer, NR4A1 shows the opposite effect in modulating glycolysis. Aberrant activated β-catenin signaling in colon cancer enhanced glycolysis; meanwhile, an NR4A1-β-catenin feed-forward loop happening in colon cancer cells proves from the side that NR4A1 may be involved in promoting glycolysis. Nonetheless, the two side effects of NR4A1 have been observed on glucose metabolism, underscoring the complex and cell depend on its metabolic regulation, demonstrating NR4A1 acts as a potential therapeutic target in malignant tumors.

Fatty acid metabolism has its particularity and university. Cells run FASN and FAO to supply necessary nutrients. NR4A1 draws increasing attention to this procedure. On the one hand, in non-cancer tissues, such as liver, muscle, and adipose, NR4A1 can alter the expression levels of fatty acid key enzymes ACC, SCD1, CPT1A, as well as transporters CD36, adiponectin receptor 2 (ADIPOR2), and Caveolin 3 (CAV3) by regulating LKB1-AMPK classic signaling pathway and its downstream SREBP1c and FAS. On the other hand, as a core hallmark of cancer, altered fatty acid synthesis is specifically important. Tumor cells drive this process to provide energy and biological materials for uncontrolled proliferation. Hence, overexpression of NR4A1 binds to TPβ to maintain the FAO-NADPH-ROS loop, leading to the suppression of cancer growth. NR4A1 has been found to bind to the NBRE or coactivator SWI/SNF complex response elements by NR4A2 or PPARγ, resulting in the change of fatty acid-related genes ACOX, CPT1M, FABP2, and FABP4 in melanoma and breast cancer. In addition, NR4A1 exhibits cholesterol regulating function by interacting with LXR in acute myeloid leukemia. All the above evidence suggests the unique and irreplaceable features in lipid metabolism; However, whether NR4A1 displays similar metabolic effects in regulating cancer metastasis, cancer stem cell phenotypes need to be deeply explored in the future.

Furthermore, MYC, mTOR, and HIF-1 are the main mediators regulating SLC transporters and glutamine synthase relative enzymes in cancers. NR4A1 is involved in glutamine metabolism through interaction with β-catenin, which further influences the expression level of Myc. In order to increase the activity of key enzymes GLS and GDH, NR4A1 induced mTOR upregulation by activating the ROS-depended AMPK signaling pathway. Similar to β-catenin, NR4A1 can stabilize and transactivate HIF-1α; meanwhile, HIF-1α bins to the promoter NR4A1 and promotes its transcription; under hypoxia, HIF-1α and NR4A1 form an interaction circuit to affect each other.

Additionally, amino acid regulation is also a function of NR4A1. It has been reported that amino acid deficiency can induce NR4A1 expression. NR4A1−/− zebrafish larva decreased total amino acids and the level of ASP, Glu, Ala, Tyr, His, Met, Pro. However, there are rare studies on tumor cells, so it could be a promising area worth exploring further. Besides the amino acid metabolism, NR4A1 also participates in tumor immunity. The COX2/NR4A1/CTNNB1 axis has been reported to increase CD34+ T effector cells, while TCR- NR4A1-β-AR system can increase metabolic reprogramming of CD8+ T-cell activation through downregulating GLUT-1 expression. Therefore, for one thing, further studies could focus on the diverse functions of NR4A1 on cancer immunity by glycolysis reprogramming. For another, maybe it can be extended the roles of NR4A1 to other aspects of tumor metabolism.

Cancer metabolism provides innovative opportunities for next-generation anticancer therapies that could be further improved using novel NR4A1 agonists or antagonists that simultaneously regulate NR4A1 and its downstream signaling pathways.

## Conclusion and perspectives

NR4A1 is a well-studied transcription factor, and recent researches focus on identifying its genomic and non-genomic effects in cancers, including melanoma (106), breast cancer (107), and colorectal cancer (31). As described above, NR4A1 exhibits important functions in cancer cell metabolic reprogramming. By regulating glucose and fatty acid-related enzymes, such as GLUT4, PEPCK1, ACC, ACLY, NR4A1 exhibits divers metabolic effects by regulating the downstream signaling pathways. NR4A1 acts as a novel application to enable tumor growth, evasion of apoptosis, migration, and invasion. However, whether NR4A1 displays the lipid metabolic functions in cancer metastasis is not fully identified. Although increasingly ligands are found to bind to, and active or inactive NR4A1, leading to cancer cell growth, apoptosis, autophagy, EMT, the study on metabolism is rare. Thus, it is an urgent need to understand the metabolic functions of NR4A1, especially how this receptor mediates fatty acid synthesis, amino acid metabolism and glutamine in tumors. The underlying mechanisms are worthy of exploring. The continued investigation of agents that can modulate NR4A1 is needed. The selective NR4A1 agonists or antagonists against cancer cell metabolism might be potential for cancer treatment.

## Author contributions

SD: Conceptualization, Methodology, Investigation, Writing-original draft. BC: Writing-review & editing. JH and XL: Writing & Revising, Supervision. All authors contributed to the article and approved the submitted version.

## Funding

This work was funded by the Natural Science Foundation of Jiangsu Province (BK20220464, BK20190938), the National Natural Science Foundation of China (No. 82004288), Project of National Clinical Research Base of Traditional Chinese Medicine in Jiangsu Province (No. JD2019SZXYB04) and Jiangsu province TCM leading talent training project (No. SLJ0211) and Natural Science Foundation of Jiangsu Province (BK20190938).

## Conflict of interest

The authors declare that the research was conducted in the absence of any commercial or financial relationships that could be construed as a potential conflict of interest.

## Publisher’s note

All claims expressed in this article are solely those of the authors and do not necessarily represent those of their affiliated organizations, or those of the publisher, the editors and the reviewers. Any product that may be evaluated in this article, or claim that may be made by its manufacturer, is not guaranteed or endorsed by the publisher.
